# Quantification of Allantoin in Yams (*Dioscorea* sp.) Using a ^1^H NMR Spectroscopic Method

**DOI:** 10.4014/jmb.2301.01009

**Published:** 2023-02-06

**Authors:** Thao Quyen Cao, Dongyup Hahn

**Affiliations:** 1 Institute of Agricultural Science and Technology, Kyungpook National University, Daegu 41566, Republic of Korea; 2 School of Food Science and Biotechnology, College of Agriculture and Life Sciences, Kyungpook National University, Daegu 41566, Republic of Korea; 3 Department of Integrative Biotechnology, Kyungpook National University, Daegu 41566, Republic of Korea

**Keywords:** Allantoin, yam, *Dioscorea*, NMR, qNMR, quantitation

## Abstract

Allantoin is an abundant component of yams and has been known as a skin protectant due to its pharmacological activities. In previous methods for allantoin determination using high-performance liquid chromatography (HPLC), the separation was unsatisfactory. We herein developed a ^1^H quantitative nuclear magnetic resonance (qNMR) method for quantification of allantoin in the flesh and peel of yams. The method was carried out based on the relative ratio of signals integration of allantoin to a certain amount of the internal standard dimethyl sulfone (DMSO_2_) and validated in terms of specificity, linearity (range 62.5-2000 μg/ml), sensitivity (limit of detection (LOD) and quantification (LOQ) 4.63 and 14.03 μg/ml, respectively), precision (RSD% 0.02-0.26), and recovery (86.35-92.11%). The method was then applied for the evaluation of allantoin in flesh and peel extracts of four different yams cultivated in Korea.

## Introduction

Nuclear magnetic resonance (NMR) spectroscopy is a key analytical technique for structures elucidation of small and macromolecules, as well as for the identification of single or multiple compounds in complex matrices [1]. In recent years, quantitative NMR (qNMR) has been well-applicable for the quantification of low molecular weight metabolites in biological fluids or food products with excellently analytical performance [2, 3]. The quantitative inaccuracy of qNMR is less than 2.0%, which is an acceptable limit for precise and accurate quantitation [4]. The ^1^H qNMR spectroscopy technique is fast and provides higher reliability on the structural prediction of the molecules [5]. Its temperature operation is low; thus preventing the degradation of thermolabile analytes [6]. In addition, the sample preparation for qNMR is of limited complexity and generally compatible with chromatography [7]. In comparison to the traditional chromatographic methods, ^1^H qNMR spectroscopy technique has not only the above certain advantages but also the possibility to simultaneously determine component structures, no need for prior isolation of the analyte present in a mixture, the possibility of simultaneous quantitative analysis of multiple target analytes in a mixture, no need for individual experimental setup, and reference of the same compound and calibrations, as well as non-invasive and non-destructive character of the method [4, 5]. Essentially, the applications of qNMR in simultaneous purity evaluation of organic molecules have great potential to advance the search for the truth behind their biological activity and to find explanations for problems that require consideration of unexpected chemical diversity due to residual complexity [5, 7].

Allantoin, a diureide of glyoxylic acid, is one of the abundant bioactive components in yams [8]. Allantoin has long been known to enhance the efficacy and desirability of various cosmetic products such as skin creams, lotions, soaps, shampoos, and lipsticks due to its antioxidative, anti-inflammatory, and moisturizing activities [9-11]. Allantoin-treated asthma groups remarkably alleviated airway inflammatory-cell infiltration as well as cytokine mRNA expression in lung tissues [12]. Additionally, allantoin has been proved to be effective on antidiabetic [13, 14], antihypertensive [15], anticancer [14], as well as on cognitive function and hippocampal neurogenesis [10].

To evaluate the contents of allantoin in yam extracts for high-quality raw materials in the development of pharmaceutical or functional food products, an assurance quantitative analytical method is needed. Several methods, including high-performance liquid chromatography (HPLC) methods, for allantoin determination, have been studied [16]. Nevertheless, no information is known in the literature on qNMR methods dealing with the analysis of allantoin in yams up to now [16-19]. Our previous study has revealed a quantitative analysis method using HPLC for allantoin identification in the peel of *Dioscorea japonica*; however, the method is limited in evaluating allantoin in the different matrices of yam [19]. The aim of this study was to develop a rapid, sensitive, and reliable ^1^H qNMR spectroscopy-based method for the allantoin quantitation in yams. The method was validated in terms of specificity, linearity, sensitivity (limit of detection (LOD) and quantification (LOQ)), precision, and recovery. The method was then used for the evaluation of allantoin in flesh and peel extracts of four *Dioscorea* species cultivated in Korea, including *Dioscorea bulbifra* L., *Dioscorea quinqueloba* Thunb, *Dioscorea batatas* Decne, and *Dioscorea esculenta* (Lour.) Burkil. These findings led to the proposal of a useful method for the analysis of allantoin obtained from various *Dioscorea* species in particular, plant extracts in general, in further discoveries of allantoin potentials as a functional biomaterial.

## Materials and Methods

### Chemical and Reference Compounds

Acetonitrile (reagent grade), water (reagent grade), and methanol (reagent grade) were purchased from J.T.Baker (USA). Trifluoroacetic acid (TFA) was purchased from Sigma-Aldrich (USA). Ethanol (extra pure grade) was purchased from Duksan Pure Chemicals Co. (Korea). Waters Alliance 2695 high-performance liquid chromatography (HPLC) (Waters, USA) which was performed on a Hector-M-carbohydrate column (250 × 4.6 mm, 5 μm, RS tech Corporation, Korea) was used for the analysis and the samples was detected by the photodiode array detector (PDA) Waters 2996. Allantoin (analytical standard grade) was purchased from Sigma-Aldrich. Dimethyl sulfoxide (DMSO-*d*_6_) was obtained from Sigma-Aldrich. Dimethyl sulfone used as internal standard (IS) was purchased from Sigma-Aldrich. IS solution was prepared in DMSO-*d*_6_ at a concentration of 1.0 mg/ml and kept at 4°C. Prior to use the IS solution was left to come to room temperature.

### Plant Material and Sample Preparation

Yams were purchased from Taesan Farm (Korea). Their tubers were washed with water and separated into flesh and peel, then dried with a freeze-dryer (Ilshinbiobase, Korea). The freeze-dried peels of four yam species were powdered, and 1 g of dried powder from each sample was sonicated with 250 ml of 50% ethanol for 30 min. The solutions were then kept at 25°C for 12 h. Afterward, the samples were filtered with filter paper (85 g/m^2^, 0.20 mm, 160 s/100 ml, Ø 110 mm, Hyundai Micro CO., Korea) and evaporated in vacuo. The obtained extracts were weighed and dissolved in methanol to inject into the HPLC system for analysis. The remaining solutions were evaporated under vacuum to dryness and used for ^1^H NMR analysis. 10.0 ± 0.2 mg of each extract was dissolved in 700 μl IS solution. The solutions containing the extract and calibrant were transferred into 5-mm NMR tubes.

### NMR Experimental Parameters

^1^H NMR spectra were recorded at 700 MHz (Bruker AVANCENeo700) with the standard qNMR conditions: temperature, 298 K; relaxation delay (D1), 60s; flip angle, 90°; acquisition time, 2.34 s; number of scans (nc), 32; and spectral width, 0‒16 ppm. Prior to Fourier transformation (FT) an exponential weighing factor corresponding to a line broadening of 0.3 Hz was applied. The spectra were phased, corrected and integrated automatically using MestReNova software. Where necessary, accurate integration was performed manually for the peaks of interest. The content of allantoin was calculated using the following equation [5]

$$\text{P[\%]=}\frac{n_{IC\cdot }Int_{A\cdot }MW_{A\cdot }m_{IC}}{n_{A\cdot }Int_{IC\cdot }MW_{IC\cdot }m_{S}}.P_{IC}$$

Where IC is the internal calibrant, A is allantoin, s is the sample, n is the number of protons, Int is integral, MW is the molecular weight, m is the mass, and P is the purity (in %).

### Calibration Curves and Validation

For the preparation of the calibration curve, the exact amount of allantoin (12.9 mg) was weighed and dissolved in 1.29 ml of IS solution. The serial dilution method was used to prepare the desired concentrations (2000, 1000, 500, 250, 125, and 62.5 μg/ml). Each amount of reference mixture was analyzed in triplicate. The quantitation was based on the integration ratio between allantoin protons at δ_H_ 8.05, 6.93, 5.80, and 5.24 ppm and dimethyl sulfone protons at δ_H_ 2.99 ppm. The method was validated following the ICH (International Council for Harmonisation of Technical Requirements for Pharmaceuticals for Human Use) guidelines “Validation of Analytical Procedures: Text and Methodology Q2(R1)” [20] for linearity, sensitivity (LOD and LOQ), accuracy, precision, and repeatability. LOD and LOQ were calculated using prepared calibration curves. The intraday precision was determined by analyzing five replicates of spiked samples at three concentration levels of allantoin (500, 250, and 125 μg/ml). The interday precision was assessed by analyzing spiked samples at three concentration levels, 500, 250, and 125 μg/ml, on five consecutive days. The precision was calculated as the relative percent standard deviation (RSD %). Accuracy was determined by recovery experiments spiking a dry extract with or without allantoin, evaluated as the relative percentage error (Er%), and measured by comparing the nominal concentration and the assayed concentration.

## Results and Discussion

**Fig. 1:**
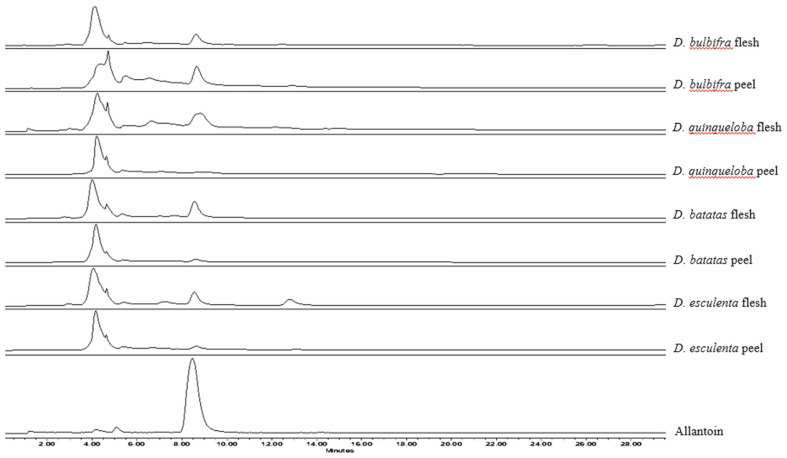
HPLC chromatograms of allantoin and yams flesh and peel extracts monitored at 235 nm.

Metabolic fingerprinting has demonstrated a significant addition to the battery of classical tools for understanding the biochemical metabolites of biological systems at a certain time [21, 22]. NMR and mass spectrometry (MS) are two analytical technologies that have arisen in metabolomics which is focused on the profiling and quantification of natural small compounds. Metabolomic studies make use mostly of hyphenated techniques which rely on chromatography separation (LC) of metabolites coupled to MS to analyze complex mixtures of extracted metabolites [23]. Although LC-MS provides a high resolution and sensitivity, LC methods generally require a long run time, metabolite separation is dependent on the chromatographic column used, detection is limited by the analytes’ ionization ability, and reference compound for peak identity and often suffers from low solubility [6]. On the contrary, ^1^H NMR is a fast analysis, highly reproducible, and robust quantitative technique [5, 22]. We first quantitated allantoin from eight yam extracts using the HPLC-photodiode array (PDA) method which was reported recently [19]. The chromatograms were obtained at 235 nm. As shown in Fig. 1, the separation was unsatisfactory. Also, the solubility of allantoin in HPLC solvents was not high. Hence, we decided to use the ^1^H qNMR method for the quantitation of allantoin in the flesh and peel of yams.

### Method Development

To develop the ^1^H NMR-based quantification technique for allantoin in yam extracts, several deuterium-labeled solvents including methanol-*d*_4_, acetone-*d*_6_, acetonitrile-*d*_3_, dimethyl sulfoxide (DMSO)-*d*_6_, deuterium oxide, as well as mixtures thereof were tested in a series of preliminary ^1^H NMR experiments as potential solubility and stability of yam extracts and allantoin. DMSO-*d*_6_ was selected for sample preparation due to the high solubility of allantoin and extracts, the good separation of NMR key resonances, and the clean baseline in the relevant spectral regions from the obtained spectra [24]. Dimethyl sulfone (DMSO_2_) was used as an internal standard (IS) for qNMR because of its solubility and stability in DMSO-*d*_6_, its non-volatility, and resulting in non-overlapping ^1^H NMR signals [5] Moreover, the ESI-MS of allantoin in the mixture with IS showed an [M-H]^+^ ion at *m/z* 157.3.

It is essential to consider the selection of NMR experimental parameters to perform the qNMR analysis due to their effects on quantitative accuracy and precision. Pulse angle and relaxation delay (D1) are the most important parameters for quantitative experiments and are closely connected [1]. In general, a 90° pulse angle was required for quantitation because of maximum intensity. In such a situation, D1 must be at least five times the longest relaxation time (T1) to guarantee full relaxation and recovery of the signal intensity [5]. Thus, D1 as 60 s and 90°pulse angle were used for the quantitative experiments.

### Validation of the ^1^H qNMR Method

**Fig. 2:**
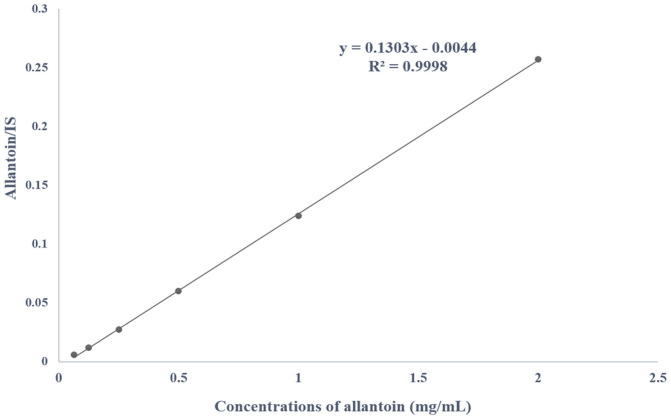
Linearity test for allantoin signals in the range 62.5‒2000 μg/ml added with a fixed quantity (0.7 mg) of IS.

The developed ^1^H qNMR method was validated in terms of specificity, linearity, sensitivity, precision, and recovery [20]. On the basis of the calibration models, the linearity of the method was confirmed for the concentrations range of 62.5‒2,000 μg/ml of allantoin added to a fixed quantity (0.7 mg) of IS (Fig. 2). The plot of the integral ratio (allantoin/IS) showed a linear trend. The linear regression equation was expressed as y = 0.1303x ‒ 0.0044; where ‘y’ and ‘x’ represent the ratio of integral of allantoin to IS and the concentrations of allantoin, respectively. The correlation coefficient of the calibration function was calculated to be 0.9998. The sensitivity parameters (LOD and LOQ) values were determined as 4.63 and 14.03 μg/ml, respectively (Table 1). Therefore, the sensitivity of the developed method was well adequate considering the working concentrations range of allantoin.

The intraday precision, expressed as RSD values ranged from 0.02 to 0.50%, as shown in Table 2. The interday precision ranged between 0.18 and 0.26% (Table 2). Accuracy was determined in recovery experiments with three different concentrations of allantoin spiked to a yam extract. The recoveries were found to be within the range of 86.35‒92.11%, with RSD% less than 0.17%. Besides that, the results for accuracy were expressed as the relative percentage error (Er%) (Table 3). The estimated accuracy values with the proposed method are within acceptable levels for allantoin. The obtained results indicate that the method could be considered accurate.

### Method Application

Yams (*Dioscorea* spp.) are economically valued herbs and are widely used for promoting health and folk medication as well [25]. Reported biological studies revealed that *D. quinqueloba* extract possesses effects on cardiovascular and inflammatory skin diseases [26, 27]. Sato *et al*. reported that *D. esculenta*-induced increase in muscle sex steroid hormone levels helps reduce insulin resistance in type 2 diabetes [28]. The powder and liquid products of *Dioscorea alata* were found to be promising in the development of blood pressure regulation foods by their antihypertensive effects on spontaneously hypertensive rats [29]. The extract and isolated metabolites of *D. batatas* were demonstrated to have wide pharmaceutical effects including antioxidant [30], anti-neuroinflammation [31], ethanol-induced gastric ulcer [32], and antidiabetic [13].

In this investigation, the developed ^1^H qNMR method was further applied for evaluating the content of allantoin in the flesh and peel extracts of four yams varieties, including *D. bulbifra*, *D. quinqueloba*, *D. batatas*, and *D. esculenta*. Among them, *D. bulbifra*, *D. quinqueloba*, and *D. batatas* were reported to have the promising antioxidant effects [33]. Our results showed that allantoin was the most abundant in the peel of *D. bulbifra* and least abundant in the *D. quinqueloba* flesh extract with contents of 62.49 and 3.30 g/kg, respectively, on the basis of weight. As shown in Table 4, allantoin was presented in both flesh and peel of all investigated *Dioscorea* species. The allantoin content of *D. bulbifra*, *D. batatas*, and *D. quinqueloba* peels is greater than that of their flesh, whereas the content of allantoin in *D. esculenta* flesh is greater than that of its peel.

As a final remark, we would like to propose a valid quantitative analysis method of allantoin in *Dioscorea* spp. using ^1^H qNMR spectroscopy-based technique. Allantoin, widely used in pharmaceutical and cosmetic applications, presents the most abundant in the peel of *D. bulbifra*. The method was validated in terms of specificity, linearity, sensitivity, precision, and recovery. The results demonstrated that the developed method was suitable for the determination of allantoin in the *Dioscorea* genus.

## Figures and Tables

**Fig. 3:**
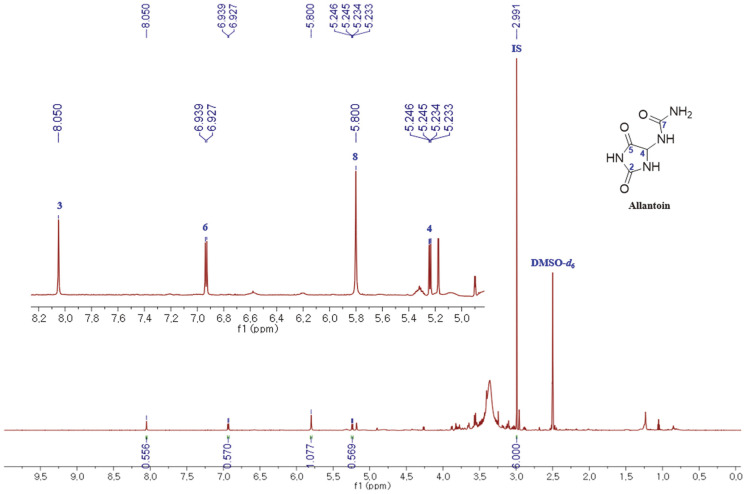
^1^H NMR spectra (700 MHz, DMSO-*d*_6_) of allantoin from *D. bulbifra* peel extract (δ 0.0‒10.0 ppm). The signals for IS, DMSO-*d*_6_, and allantoin chemical markers have been assigned.

**Table 1:** Linearity of allantoin determination in yams.

Ratio	Regession eq	Correlation coeff, *r*^2^	Linearity range (μg/ml)	LOD (μg/ml)	LOQ (μg/ml)
Allantoin/IS	*y* = 0.1303*x*-0.0044	0.9998	62.5‒2000	4.63	14.03

**Table 2:** Precision data of allantoin in yams (intraday (*n* = 5 on each day) and interday (*n* = 5)).

Ratio	Theoretical concentration (μg/ml)	RSD (%)					

		Day 1	Day 2	Day 3	Day 4	Day 5	Days 1‒5
Allantoin/IS	125	0.5	0.17	0.07	0.15	0.14	0.26
	250	0.12	0.08	0.15	0.09	0.09	0.16
	500	0.14	0.05	0.06	0.02	0.05	0.18

**Table 3:** Accuracy data of allantoin determination in yams (*n* = 5).

Ratio	Relative percentage error (Er%)			Recovery (% ± RSD)		

	62.5 (μg/ml)	125 (μg/ml)	500 (μg/ml)	62.5 (μg/ml)	125 (μg/ml)	500 (μg/ml)
Allantoin/IS	‒9.76	‒7.89	‒13.65	90.24 ± 0.17	92.11 ± 0.15	86.35 ± 0.11

**Table 4:** qNMR results for allantoin in yams (*n* = 3).

Content (g/ kg ± RSD)								

	Flesh				Peels			

	*D. bulbifra*	*D. batatas*	*D. esculenta*	*D. quinqueloba*	*D. bulbifra*	*D. batatas*	*D. esculenta*	*D. quinqueloba*
Allantoin	19.14 ± 1.71	22.01 ± 2.38	16.12 ± 0.84	3.30 ± 2.05	62.49 ± 0.49	25.35 ± 0.36	13.55 ± 0.51	5.19 ± 1.33
